# Schnyder Corneal Dystrophy in a Saudi Arabian Family with Heterozygous *UBIAD1* Mutation (p.L121F)

**DOI:** 10.4103/0974-9233.75890

**Published:** 2011

**Authors:** Huda Al-Ghadeer, Jawahir Y. Mohamed, Arif O. Khan

**Affiliations:** King Khaled Eye Specialist Hospital, Riyadh, Saudi Arabia; 1 Department of Genetics, King Faisal Specialist Hospital and Research Center, Riyadh, Saudi Arabia; 2 King Khaled Eye Specialist Hospital and Department of Genetics, King Faisal Specialist Hospital and Research Center, Riyadh, Saudi Arabia

**Keywords:** Cholesterol, Saudi Arabia, Schnyder Corneal Dystrophy, *UBIAD1*

## Abstract

Schnyder corneal dystrophy is a rare dominant disorder mostly reported in Western and occasionally Asian populations. This report documents the condition in an affected family from the historically isolated Arabian Peninsula. A child and her mother had central crystalline keratopathy consistent with Schnyder corneal dystrophy. Diagnostic *UB1AD1* testing revealed a known point mutation (c.361C>T, p.L121F) in both individuals. Available asymptomatic family members had normal ophthalmic examinations and did not have the mutation. Blood lipid profiles for the two patients revealed mildly elevated total cholesterol and low-density lipoproteins. This report documents Schnyder corneal dystrophy on the Arabian Peninsula and further confirms its relationship with heterozygous *UB1AD1* missense mutation.

## INTRODUCTION

Schnyder corneal dystrophy (SCD; Online Mendelian Inheritance in Man #121800) is a rare autosomal dominant dystrophy characterized by central anterior stromal cholesterol deposition in an annular or disciform distribution that can be seen as early as the first decade of life.^1^,^2^ With time, arcus lipoides and mid-peripheral haze develop.^1^,^2^ Although clinically visible crystals have often been considered a classical part of the phenotype, they are only present 50% of the time and thus are not essential for the diagnosis.^1^–^3^ Genu valgum and dyslipidemia are potential associated systemic features.^1^,^2^ If crystals are visually symptomatic, laser ablation is successful for superficial disease while penetrating corneal surgery is an option when diffuse stromal haze develops.

Since heterozygous missense mutation in the highly conserved gene *UBIAD1* was first associated with the disorder,^4^,^5^ at least 21 different missense mutations have been reported to underlie SCD in 40 mostly Western and Asian families.^4^–^11^ We describe an affected family from the Arabian Peninsula and document that heterozygous missense *UBIAD1* mutation segregated with the phenotype.

## CASE REPORT

**Figure 1:**
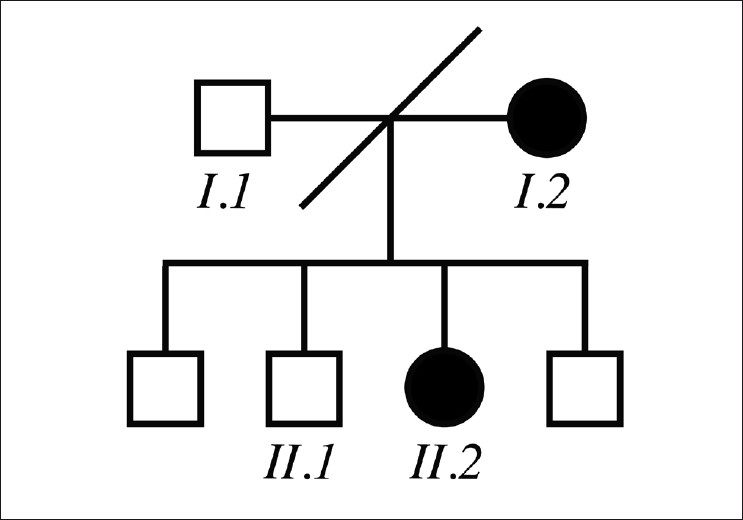
(Pedigree) The family was not consanguineous and the parents were divorced. Other than the mother and daughter, there was no other known family member with ocular signs or symptoms

Institutional review board consent was obtained for this report. A mother and her daughter were the only two members of a nuclear family with ocular complaints [Figure 1]. All available family members were seen for clinical examination, blood testing including genetic analysis, and genetic counseling. The symptomatic mother, the asymptomatic father, the asymptomatic daughter, and one asymptomatic son participated fully after giving informed consent. The two other asymptomatic sons only participated in clinical examinations.

### Clinical features

#### Affected patients (the mother and her daughter)

**Figure 2:**
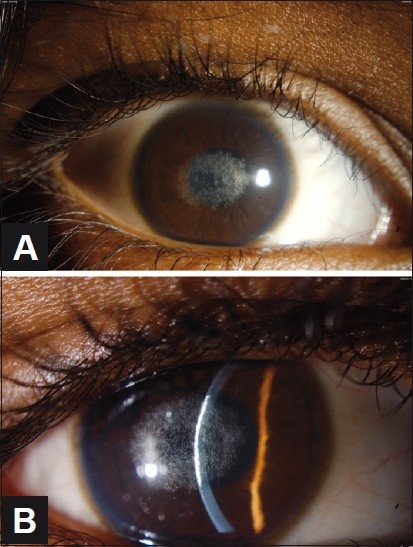
(A and B) (Proband) Slit-lamp examination reveals symmetric central anterior stromal corneal crystals without corneal opacity or haze in both corneas. The right eye is shown

A 10-year-old girl had no visual complaints but had abnormal eye color noted for years. Uncorrected visual acuity was 20/30 in the right eye and 20/25 in the left eye. Ophthalmic examination was significant for central anterior stromal corneal crystals without corneal opacity or haze [Figure 2]. Corneal sensation was normal. Anterior and posterior chamber structures were within normal limits, as were pupillary reactions and cycloplegic refraction. There was no xanthelasma or genu valgum. The fasting plasma level of total cholesterol was marginally elevated (5.4 mmol/L; normal range, 0.0-5.2 mmol/L) as was the fasting level of low density lipoproteins (3.44 mmol/L; normal range, 0.0-3.20 mmol/L). Results of urinalysis, blood cell counts, serum electrolytes, serum proteins, fasting triglycerides, and high density lipoproteins were within normal limits.

**Figure 3:**
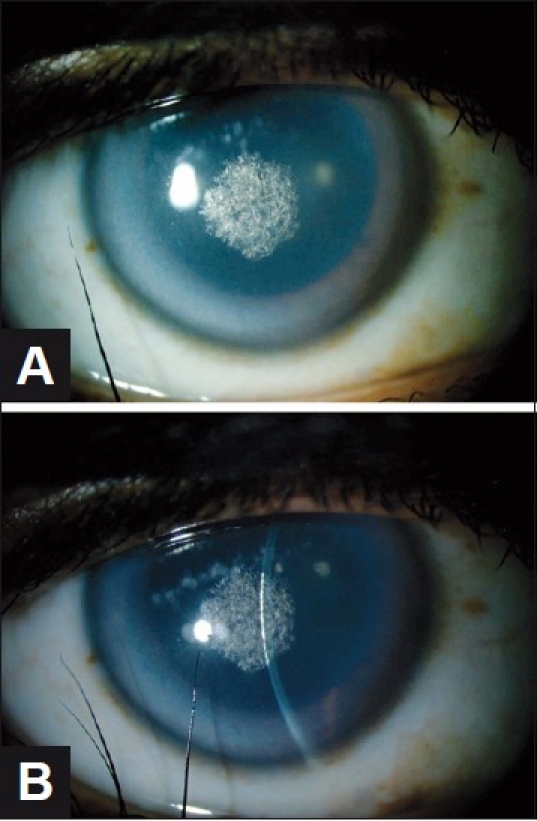
(A and B) (Affected mother) Slit-lamp examination reveals symmetric central anterior stromal opacity with crystalline deposits and prominent arcus senilis in both corneas. The right eye is shown

The proband’s 35-year-old mother was a Saudi woman who complained of bilateral painless gradual reduction of vision over the previous 10 years. She denied any medical problems. Uncorrected visual acuity was 20/30 for both eyes. Ophthalmic examination was significant for central anterior stromal opacity with crystalline deposits and prominent arcus lipoides in both corneas [Figure 3]. Corneal sensation was normal. Anterior and posterior chamber structures were within normal limits, as were pupillary reactions and refraction. There was no xanthelasma or genu valgum. The fasting plasma level of total cholesterol was marginally elevated (5.4 mmol/L; normal range, 0.0-5.2 mmol/L) as was the fasting level of low density lipoproteins (3.44 mmol/L; normal range, 0.0-3.20 mmol/L). Results of urinalysis, blood cell counts, serum electrolytes, serum proteins, fasting triglycerides, and high density lipoproteins were within normal limits.

#### Asymptomatic relatives

The proband’s ex-husband and three other children from their marriage had unremarkable ophthalmic examinations and no medical disease. There was no family history of stroke or sudden death of undetermined etiology. Ophthalmic examination, with particular attention to corneal findings, was unremarkable.

### Genetic features

After informed consent, genomic DNA was extracted from the affected child, her affected mother, her father, and her oldest brother (5 ml of blood in EDTA from each individual). On the affected child’s sample, all *UBIAD1* coding exons as well as their flanking intronic sequences were amplified by polymerase chain reaction (PCR) on MyCycler (Bio-Rad Laboratories Inc., Hercules, CA, USA) (primer sequences and PCR conditions are available upon request). On the other three samples, a targeted PCR amplification of the mutation-containing fragment was carried out. Amplicons were purified then bidirectionally sequenced on ABI 3730xl DNA Analyzer (Applied Biosystems Inc., Carlsbad, CA, USA). Sequence analysis was performed using Lasergene (DNAStar Inc., Madison, WI, USA).

**Figure 4:**
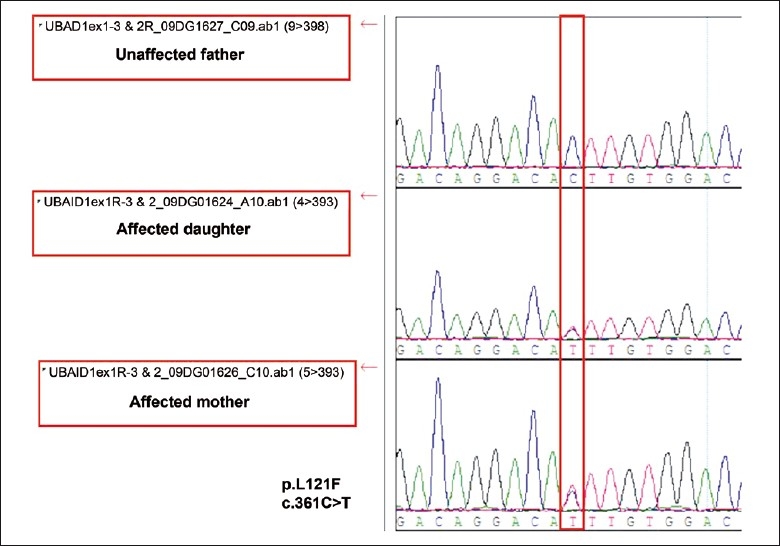
(DNA chromatogram): UBAID1 sequencing revealed p.L121F heterozygous mutation in the affected mother and daughter only

A previously reported heterozygous point mutation c.361C>T (p.L121F)^7^ was detected in the affected child and the affected mother but not in the asymptomatic father or the asymptomatic brother [Figure 4].

## DISCUSSION

To the best of our knowledge, all genetically tested SCD cases published to date have had heterozygous missense *UBIAD1* mutation.^4^–^11^ These cases have been from Europe, North America, Southeast Asia, and from one family from Egypt [Table 1]. Documentation of missense *UBIAD1* mutation in this affected Saudi Arabian family is further evidence of the phenotype’s specificity for heterozygous missense mutation in *UBIAD1*.

*UBIAD1* is a highly conserved gene across vertebrates and invertebrate orthologs.^4^,^5^,^11^ Residues 58-333 contain a prenyltransferase domain for which the bacterial archetype is bacterial protein UbiA.^4^,^5^,^11^ All reported mutations associated with SCD to date fall within this domain.^4^–^11^ The protein may play a direct role in intracellular cholesterol biochemistry and/or prenylation of other proteins regulating cholesterol transport and storage. There does not appear to be a genotype/phenotype correlation for specific *UBIAD1* mutations, although residue 102 may be a hotspot for mutation. Sixteen out of 40 previously reported families^4^–^11^ without known relation had p.N102S mutation, while the next highest number of presumably unrelated families had a specific mutation of 3/40 for p.G177R [Table 1]. Studies of UBIAD1 homology, localization, and structure suggest an underlying pathology of loss-of-function for the missense mutations reported in the gene to date.^11^

**Table 1:** Prior reported mutations

Ethicity of families	Mutation	^#^Families	Reference
Irish-French Canadian	A97T	1	11
Chinese	G98S	1	9
Multiple*	N102S	16	4,5,6,7,11
French-British Canadian	D112N	1	11
East Indian	D112G	1	5
American	D118G	1	7
Canadian; African-American	R119G	2	5, 6
British; American	L121F	2	7
Egyptian	L121V	1	6
Native American	V122E	1	11
Finnish	V122G	1	11
German	S171P	1	7
Japanese	Y174C	1	8
Scottish; Hungarian-American	T175I	2	5,7
American; Taiwanese; Kosovar	G177R	3	4,7
Japanese	K181R	1	8
German-American	G186R	1	7
Chinese-Taiwanese	L188H	1	11
Canadian	N232S	1	5
Japanese	N233H	1	8
African-American	D236E	1	7
	21 mutations	40 families	

* 7 American; 2 German; 2 British; Irish; Italian; Czech; Chinese-Taiwanese; Japanese

Both genu valgum and hypercholesterolemia have been described as potential systemic associations of SCD that have no relationship with the degree of corneal involvement.^1^,^2^ However, it is unclear how strong these potential associations are because of the following: (1) these two findings occur frequently in the general population while SCD is rare; (2) not all SCD patients have been assessed for these findings; (3) those that have been assessed were not assessed in a standardized fashion.^1^,^2^ Genu valgum was described in 5/144 patients (4%) in one series of predominantly clinically diagnosed cases (five individuals from three families).^1^ Hypercholesterolemia has been described in up to 66% of patients in different clinical series.^2^ Our patients did not have genu valgum but did have evidence for dyslipidemia. The fact that *UBIAD1* likely plays a role in cholesterol biochemistry indicates that the association with hypercholesterolemia is real.

In summary, this report together with the current published literature suggests that SCD is specific for heterozygous missense *UBIAD1* mutation. As only missense mutations have been associated with SCD to date, other types of *UBIAD1* mutations may underlie phenotypes that have not yet been associated with the gene.
